# Supplementary material for: The intensification of Arctic warming as a result of CO2 physiological forcing
Source: Nat Commun. 2020 Apr 29;11:2098. doi: 10.1038/s41467-020-15924-3 (PMC7190732; doi:10.1038/s41467-020-15924-3)
Supplement: Supplementary file 1 — Supplementary information [file 41467_2020_15924_MOESM1_ESM.pdf]

# Supplementary Information for

## The intensification of Arctic warming as a result of $CO_2$ physiological forcing

So-Won Park[1], Jin-Soo Kim[2,3,*], Jong-Seong Kug[1,*]

[1] Division of Environmental Science and Engineering,

Pohang University of Science and Technology (POSTECH), Pohang, South Korea

[2] School of GeoSciences,

University of Edinburgh, Edinburgh, United Kingdom

[3] National Centre for Earth Observation,

University of Edinburgh, Edinburgh, United Kingdom

*Corresponding author. Email: jskug@postech.ac.kr and Jinsoo.Kim@ed.ac.uk

**Supplementary Note 1: Weakness of Dynamic Global Vegetation Model (DGVM)**

GFDL-ESM2M, HadGEM2-ES and MPI-ESM-LR were coupled with dynamic vegetation modules (Supplementary Table 2), which simulate shifts in the land cover and its associated biogeochemical and hydrological processes as a response to climate change. DGVMs are currently the best available ways to represent vegetation dynamics used in global scale studies and also the coupling of DGVMs and GCMs provide an opportunity to assess the vegetation–atmosphere interactions in climate simulations[1]. However, several studies have consistently suggested that dynamic vegetation models have serious weaknesses in reproducing the observed vegetation and albedo dynamics in the Northern hemisphere and thereby contribute to bias in climate simulations[2–4]. The vegetation models overestimate the mean and trend of LAI particularly in the boreal forest and also simulate the longer growing season compared to the observations[2–4]. In addition, nutrients cycles, such as nitrogen that limits the capacity of the plants growth and is tightly coupled with the carbon cycle, are not fully integrated and poorly represented in DGVMs[1]. Nevertheless, the incorporation of dynamic vegetation modules appears to have little effect on intermodel differences of projection in LAI[5]. There also remain the issues that the impact of the dynamic vegetation modules on a coupled DGVM–GCM experiment depends on the strength of the land–atmosphere coupling and this coupling strength can vary widely between models[1].

**Supplementary Note 2: Uncertainties in the projection of physiological forcing**

While the reduction in plant transpiration under elevated $CO_2$ levels was observed within a large number of species throughout the experiments, in some species there was no evidence for this[6,7]. The stomatal conductance parameterization, used in the ESMs, is rather static and semi-empirical, and thus does not simulate realistic stomatal behavior of various plants[8,9]. Moreover, current ESMs have difficulty in simulating the observed response of LAI to higher $CO_2$ concentrations, which additionally contributes to the uncertainty[10]. Thus, there is an uncertainty in terms of the quantitative prediction of the magnitude of Arctic warming due to the uncertainty in the projection of physiological forcing, despite the robust intermodel relationship between the remote effect of physiological forcing and the Arctic warming.

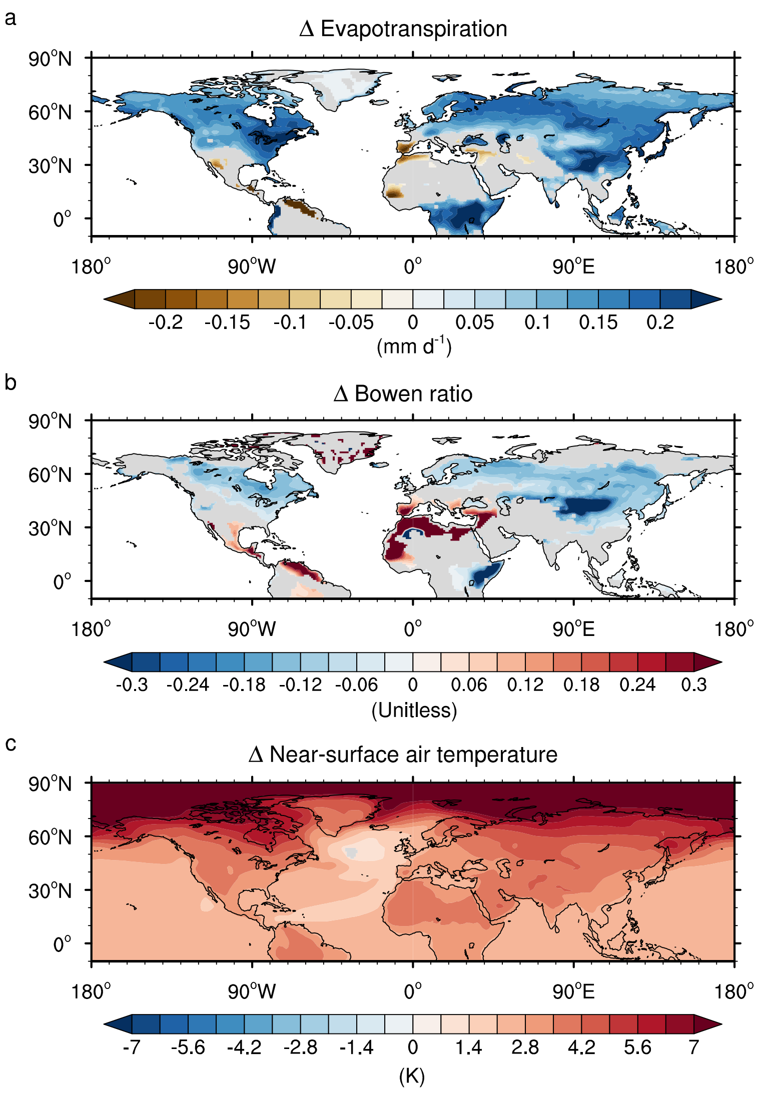


**Supplementary Figure 1 | Change in the annual mean evapotranspiration, Bowen ratio and near-surface air temperature resulting from $CO_2$ radiative forcing. a–c,** Multi-model mean change of the annual mean evapotranspiration (**a**), Bowen ratio (sensible heat flux/latent heat flux) (**b**), and near-surface air temperature (**c**) resulting from $CO_2$ radiative forcing. Only significant values at the 95% confidence level based on a bootstrap method are shown.

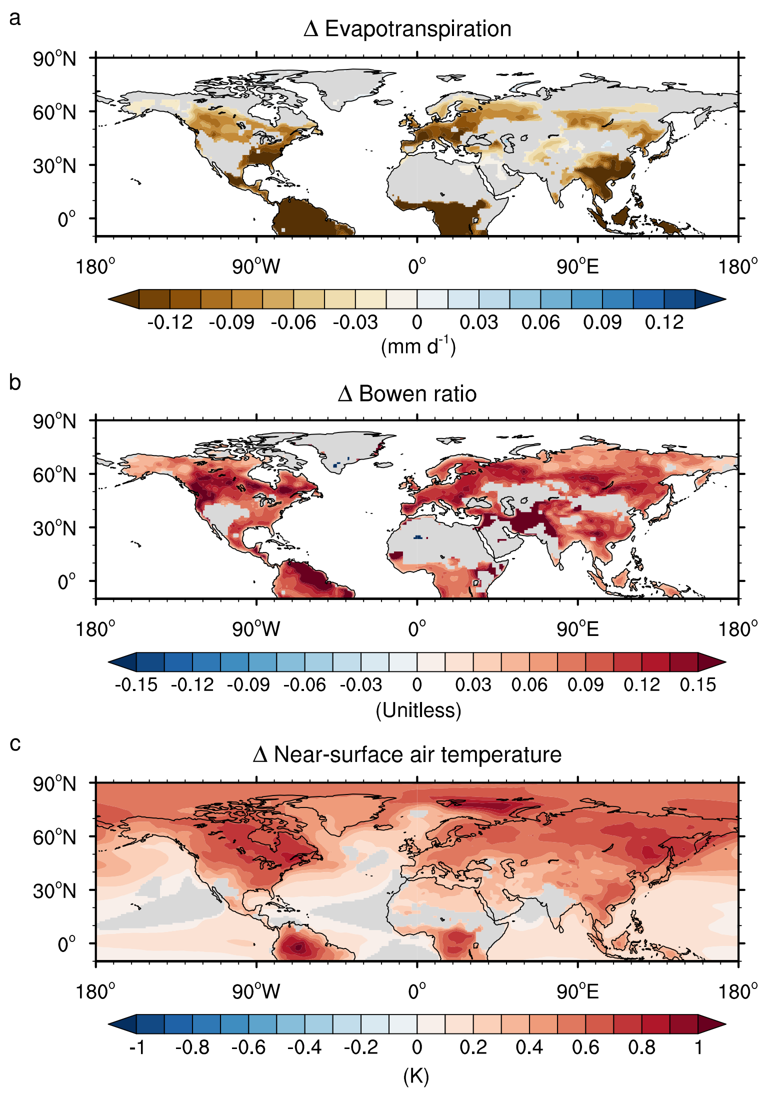


**Supplementary Figure 2 | Change in the annual mean evapotranspiration, Bowen ratio and near-surface air temperature resulting from only $CO_2$ physiological forcing. a–c,** Multi-model mean change of the annual mean evapotranspiration (**a**), Bowen ratio (sensible heat flux/latent heat flux) (**b**), and near-surface air temperature (**c**) resulting from only-$CO_2$ physiological forcing excluding a nonlinear interaction between physiogical forcing and radiative forcing. Only significant values at the 95% confidence level based on a bootstrap method are shown.

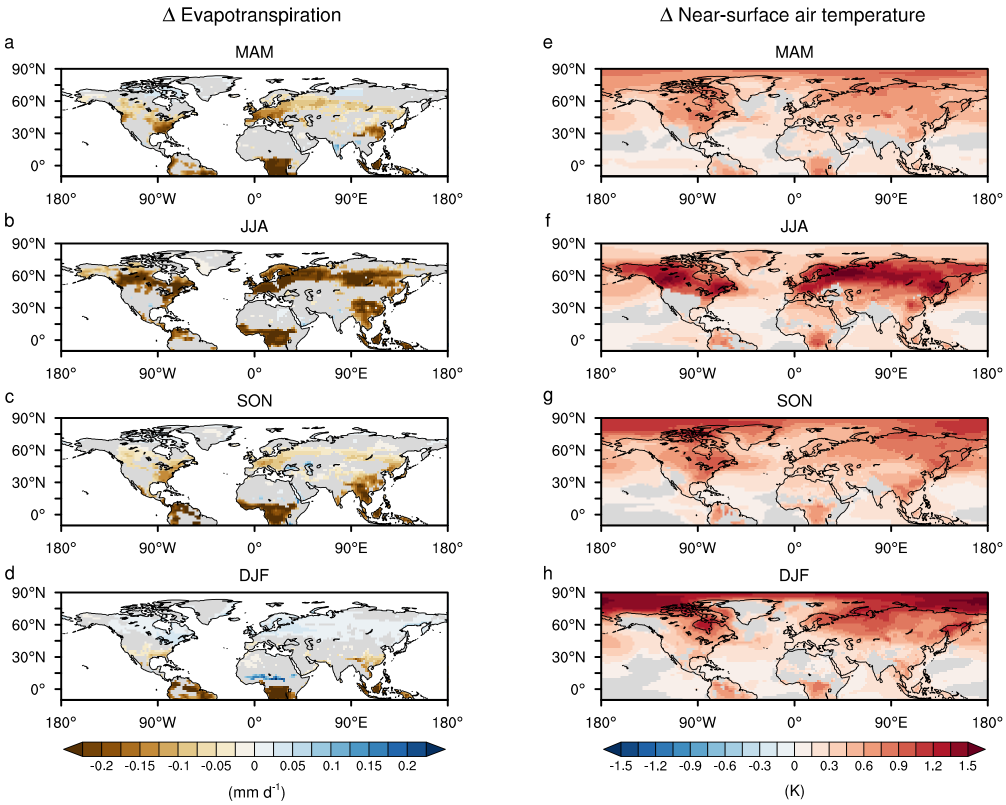


**Supplementary Figure 3 | Seasonal change in the evapotranspiration and near-surface air temperature resulting from $CO_2$ physiological forcing. a-d,** Multi-model mean change of evapotranspiration in March–April–May (MAM) (**a**), June–July–August (JJA) (**b**), September–October–November (SON) (**c**), and December–January–February (DJF) (**d**). **e-h,** Multi-model mean change of near-surface air temperature in MAM (**e**), JJA (**f**), SON (**g**), and DJF (**h**). Only significant values at the 95% confidence level based on a bootstrap method are shown.

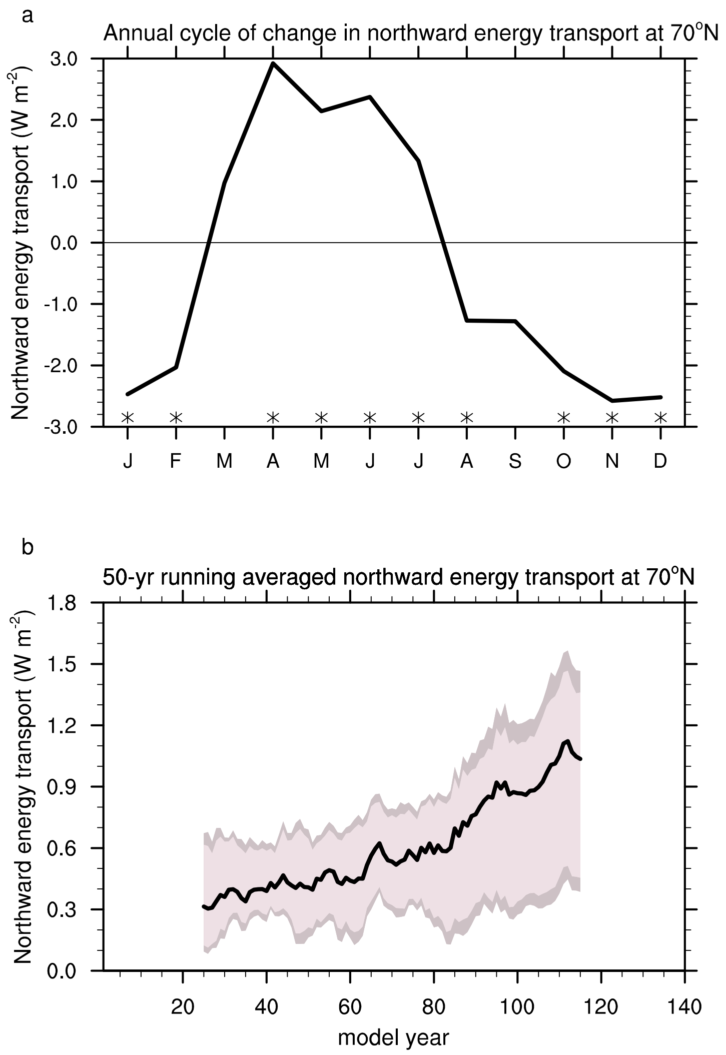


**Supplementary Figure 4 | Change in atmospheric northward energy transport ($NHT_{ATM}$) at 70°N resulting from $CO_2$ physiological forcing. a,** Annual cycle of multi-model mean change of atmospheric energy convergence into the Arctic basin. Black line indicates the change in $NHT_{ATM}$ resulting from $CO_2$ physiological forcing averaged over the final 50 years of the simulations. Black stars indicate significant months for the change in $NHT_{ATM}$ at 90% confidence levels based on a bootstrap method. **b,** Time series of multi-model mean change in atmospheric northward energy transport at 70°N during April–September. $NHT_{ATM}$ was filtered with a 50-year moving average. Light pink shades indicate 90% confidence levels and deep pink shades represent 95% confidence levels based on a bootstrap method.

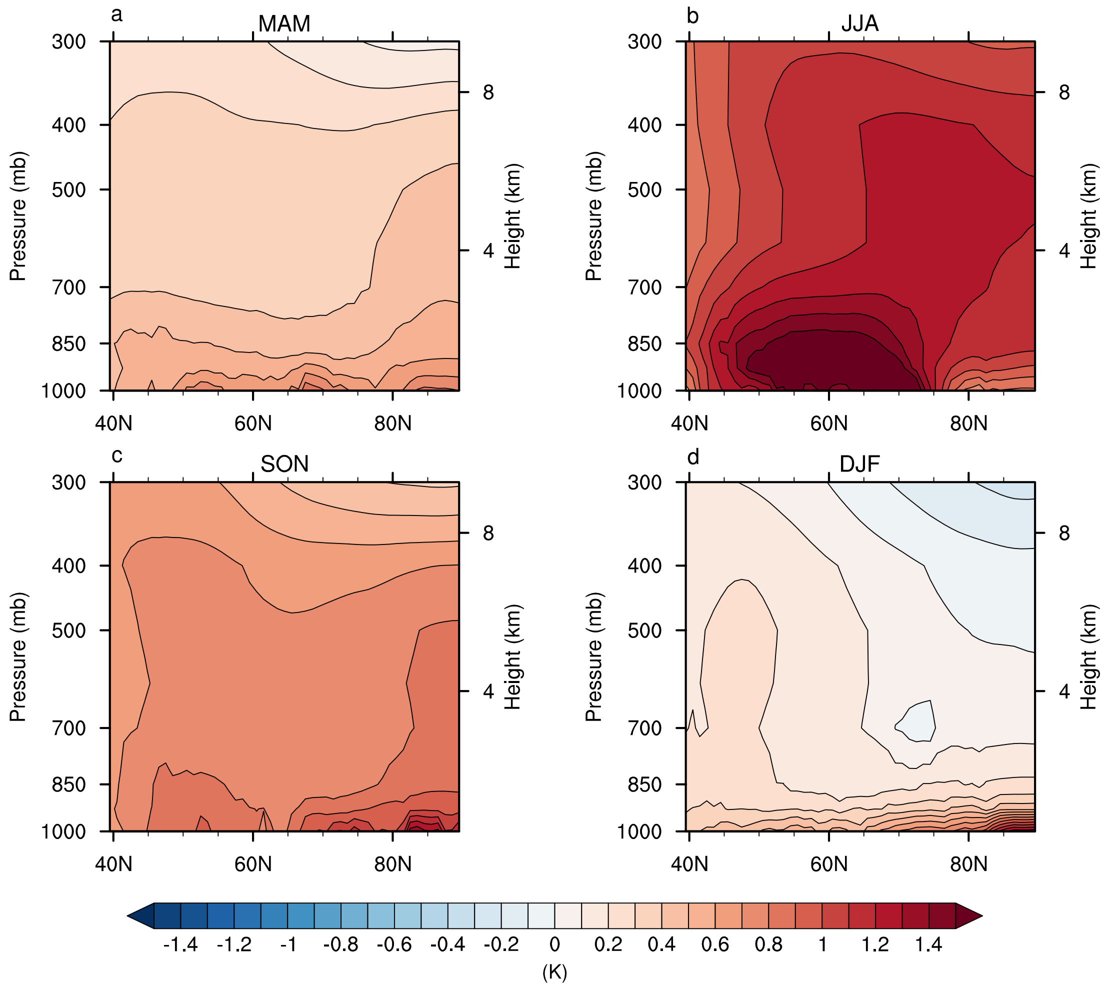


**Supplementary Figure 5 | Seasonal change in vertical structure of atmospheric warming resulting from $CO_2$ physiological forcing. a-d,** Vertical structure of zonally averaged temperature change resulting from $CO_2$ physiological forcing during March–April–May (MAM) (**a**), June–July–August (JJA) (**b**), September–October–November (SON) (**c**), and December–January–February (DJF) (**d**) in the Northern hemisphere (40°–90°N) from CMIP5 multi-model ensemble.

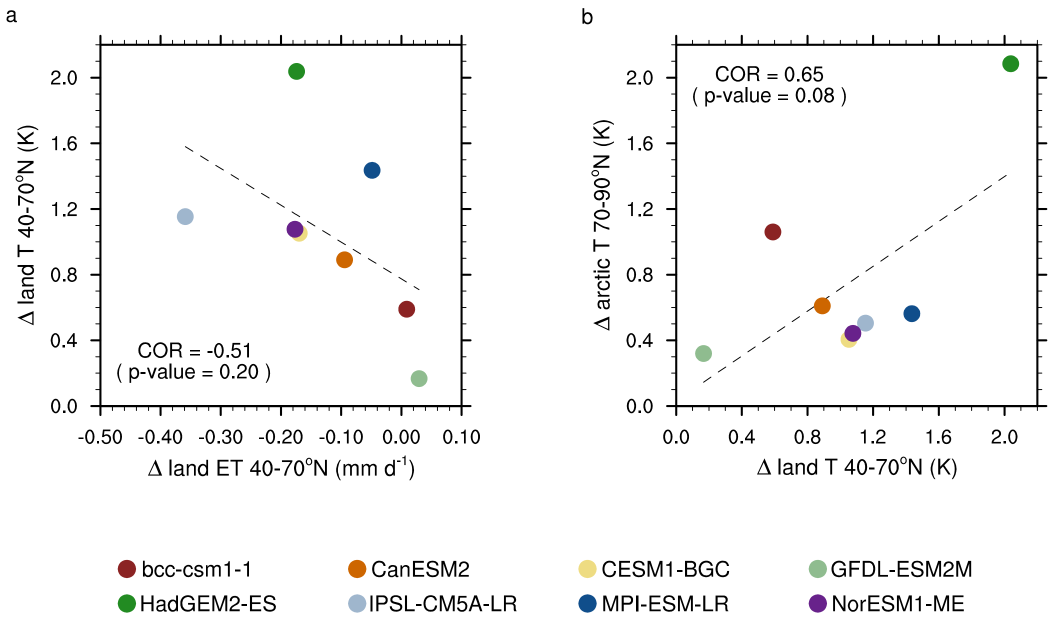


**Supplementary Figure 6 | Impacts of $CO_2$ physiological forcing on the surface warming in the continent (40°–70°N) and the Arctic (70°–90°N). a,** Scatterplot of change in evapotranspiration versus near-surface air temperature over continental regions (40°–70°N) resulting from $CO_2$ physiological forcing during summer. **b,** Scatterplot of change in surface air temperature over continents (40°–70°N) during summer versus changes in annual mean near-surface air temperature over Arctic region (70°–90°N). All values are area-weighted averages of CMIP5 multi-model ensemble.

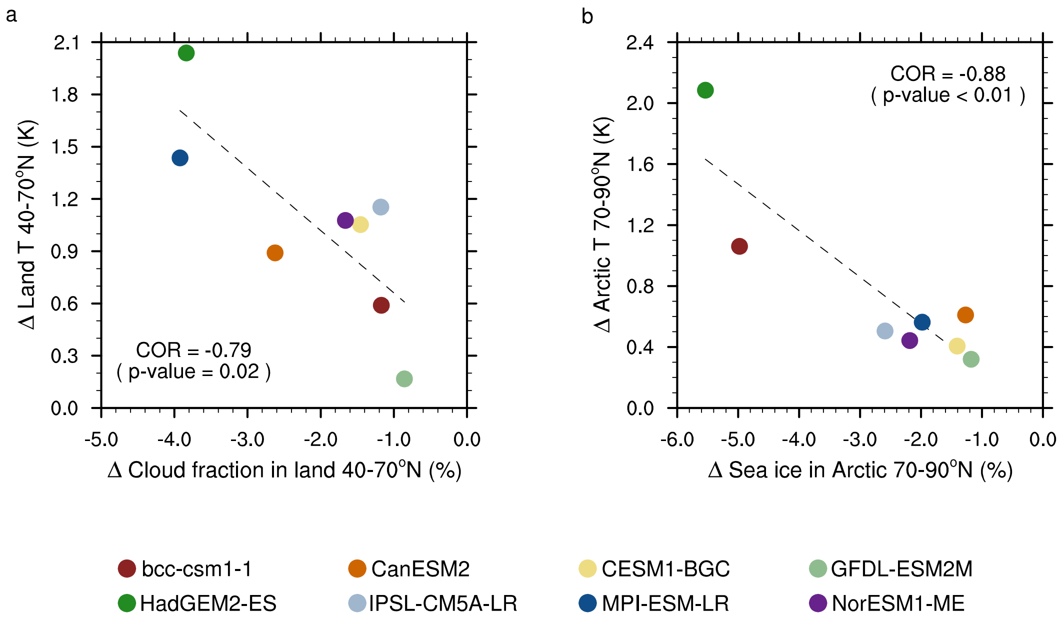


**Supplementary Figure 7 | Impacts of local feedbacks resulting from $CO_2$ physiological forcing on the surface warming in the continent (40°–70°N) and the Arctic (70°–90°N). a,** Scatterplot of change in the total cloud fraction versus near-surface air temperature over continent (40°–70°N) resulting from $CO_2$ physiological forcing during summer. **b,** Scatterplot of annual mean change in the sea ice fraction versus near-surface air temperature over Arctic region (70°–90°N) resulting from $CO_2$ physiological forcing. All values are area-weighted averages of CMIP5 multi-model ensemble.

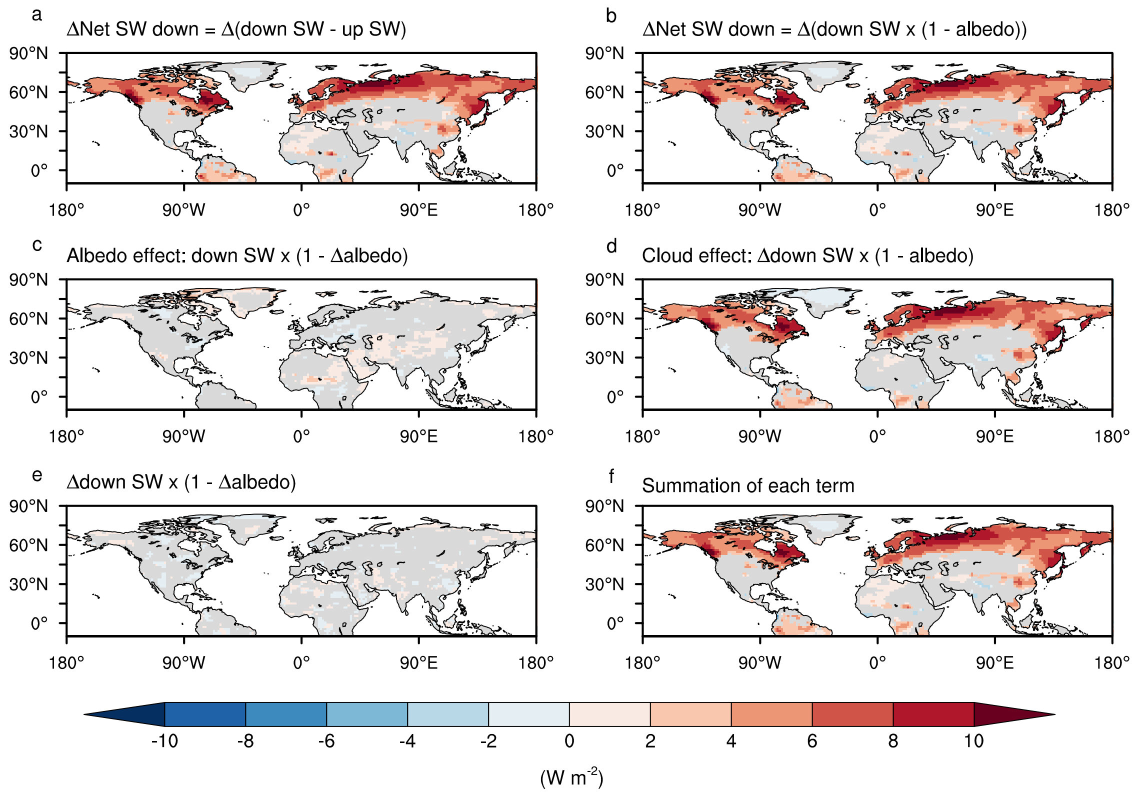


**Supplementary Figure 8 | Relative contributions to change in net surface SW absorption in summer. a,b,** Multi-model mean change of net SW radiation, calculated from Δ(Down SW – Up SW) **(a)** and Δ(down SW×(1 - albedo)) **(b)**, can be decomposed based on Eq (1): ΔNet SW down = down SW×(1 - Δalbedo) + Δdown SW×(1 - albedo) + Δdown SW×(1 - Δalbedo) (1). **c-e,** Each term in eq. **(1)** plotted as relative contributions to change of net SW radiative flux from the $CO_2$ fertilization-induced albedo effect, down SW×(1 - Δalbedo) **(c)**, cloud effect resulting from decreased stomatal conductance, Δdown SW×(1 - albedo) **(d)**, and residual effect, Δdown SW×(1 - Δalbedo) **(e)**. **f,** Sum of changes in net SW absorption based on each term in Eq. **(1)**. Only statistically significant values at the 95% confidence level based on a bootstrap method are shown. (positive: downward)

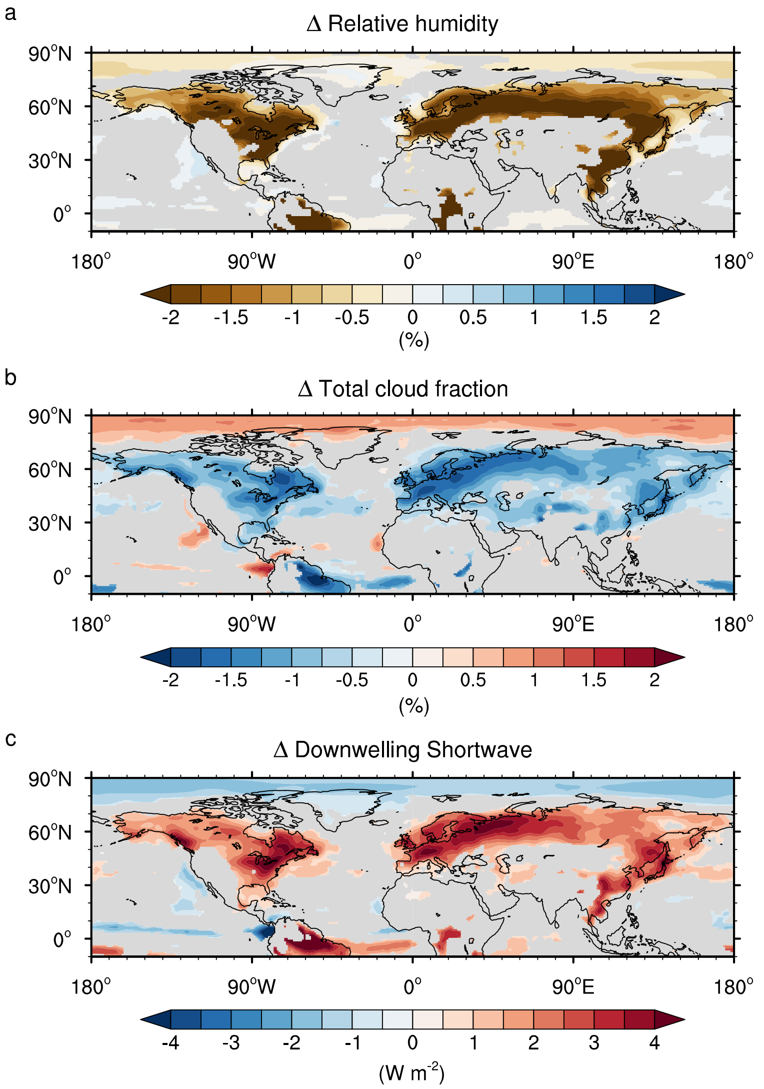


**Supplementary Figure 9 | Change in the annual mean relative humidity, total cloud fraction and downwelling shortwave radiation resulting from $CO_2$ physiological forcing. a–c,** Multi-model mean change of the relative humidity (**a**), total cloud fraction (**b**), and downwelling shortwave radiative flux (**c**) resulting from $CO_2$ physiological forcing. Only significant values at the 95% confidence level based on a bootstrap method are shown.

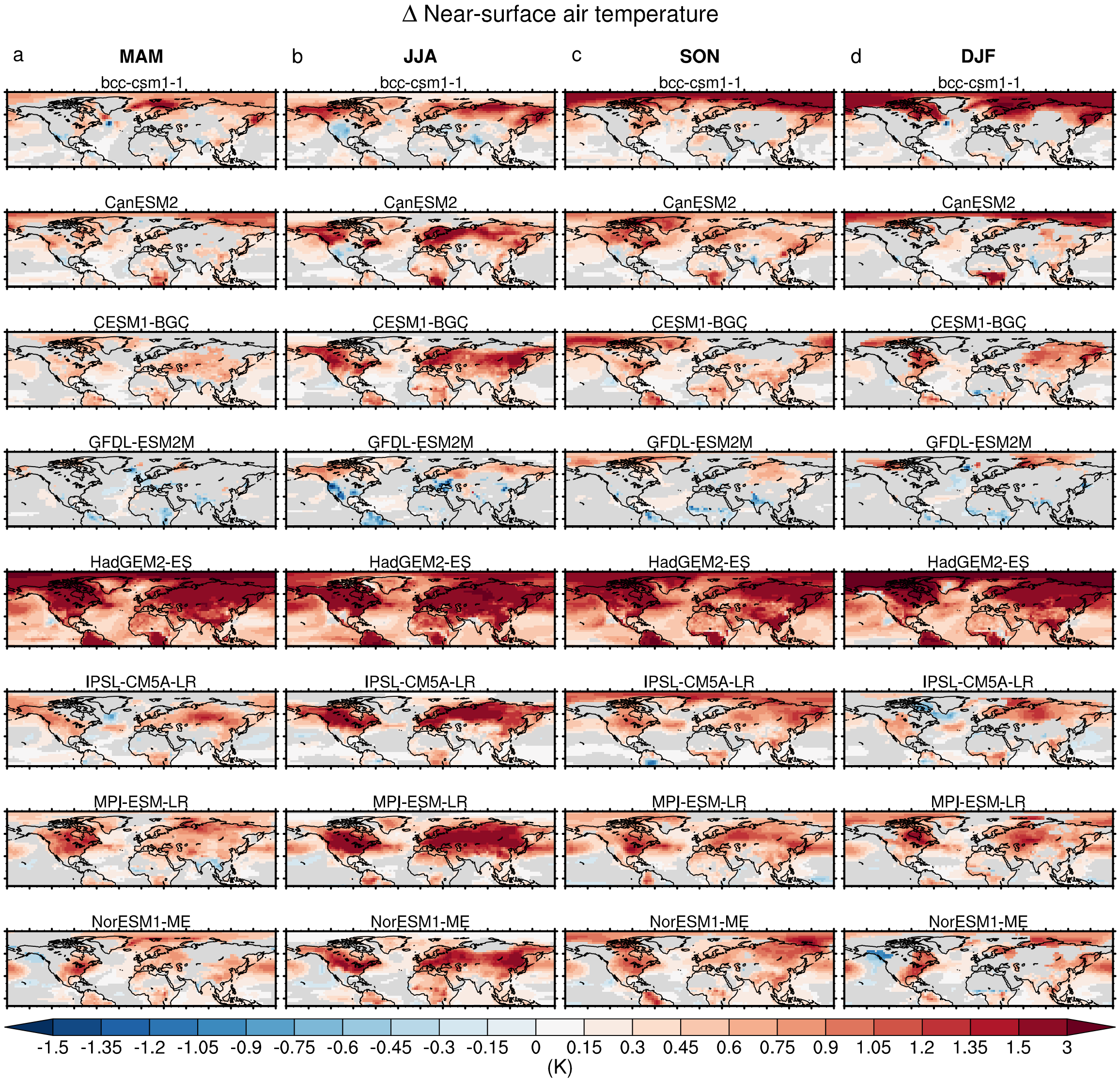


**Supplementary Figure 10 | Seasonal change in the near-surface air temperature resulting from $CO_2$ physiological forcing from CMIP5 ESMs. a–d,** Change of near-surface air temperature in March–April–May (MAM) (**a**), June–July–August (JJA) (**b**), September–October–November (SON) (**c**), and December–January–February (DJF) (**d**) from each individual model. Only significant values at the 90% confidence level based on a bootstrap method are shown.

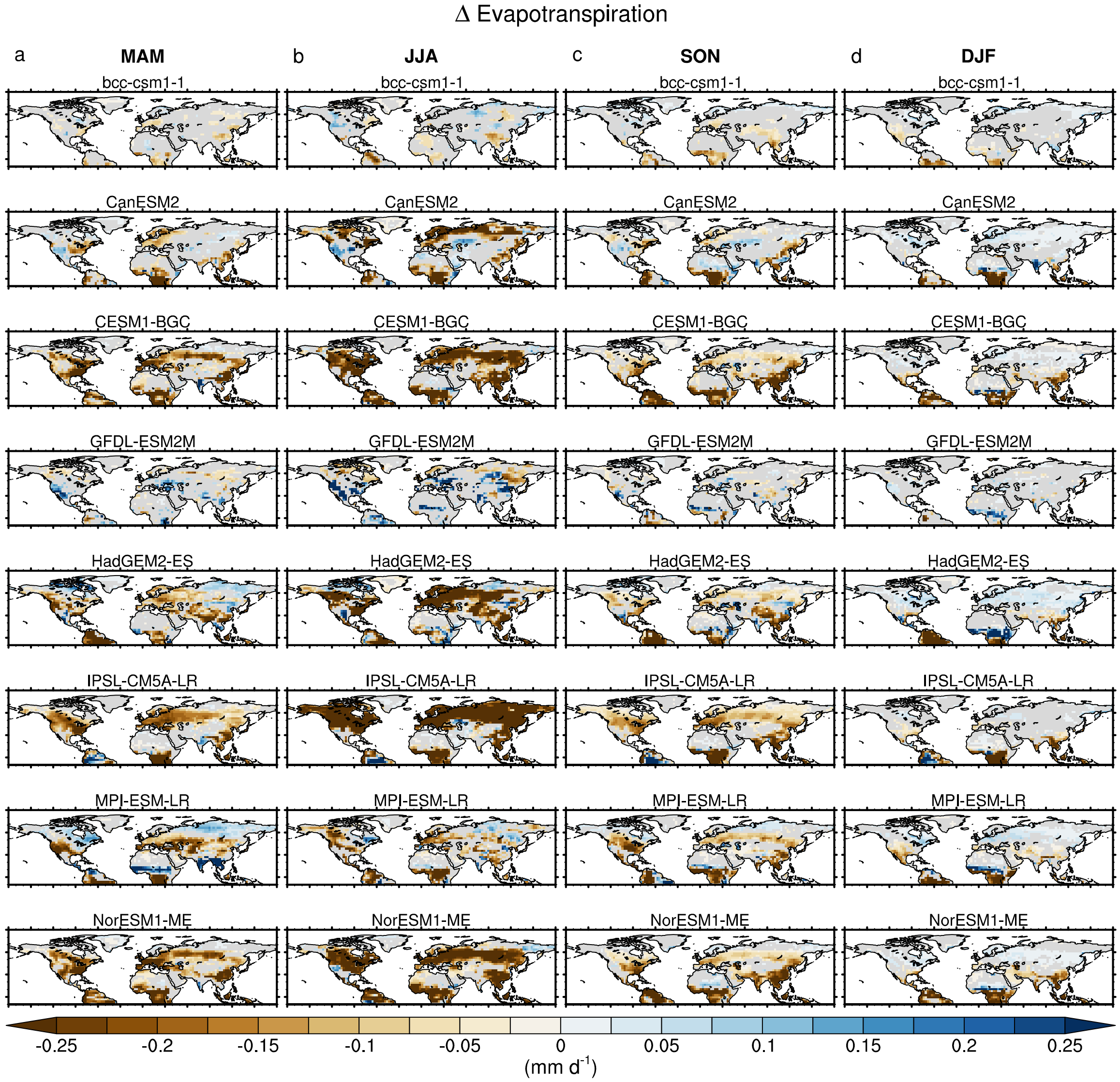


**Supplementary Figure 11 | Seasonal change in the evapotranspiration resulting from $CO_2$ physiological forcing from CMIP5 ESMs. a–d,** Change of evapotranspiration in March–April–May (MAM) (**a**), June–July–August (JJA) (**b**), September–October–November (SON) (**c**), and December–January–February (DJF) (**d**) from each individual model. Only significant values at the 90% confidence level based on a bootstrap method are shown.

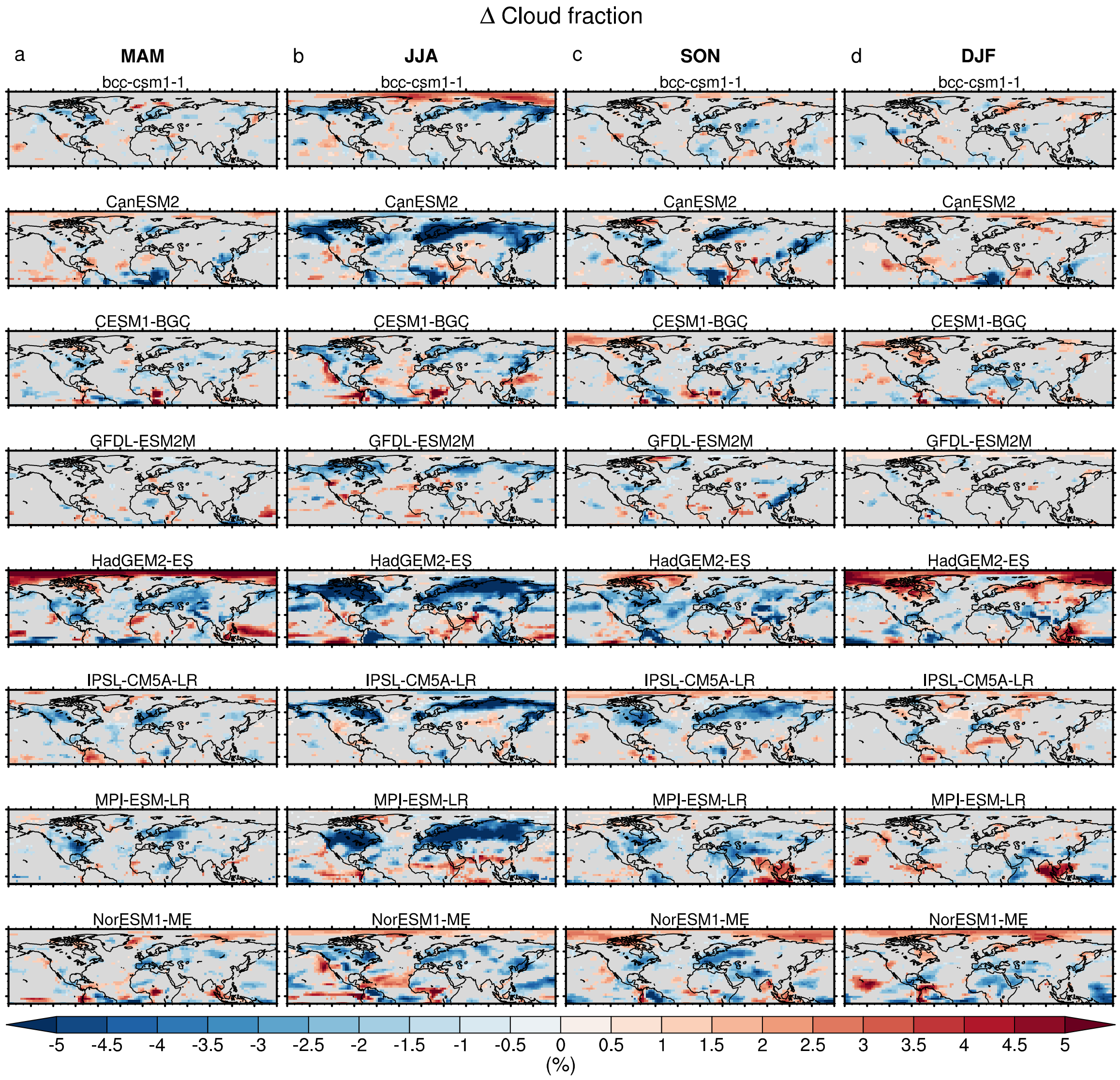


**Supplementary Figure 12 | Seasonal change in the total cloud fraction resulting from $CO_2$ physiological forcing from CMIP5 ESMs. a–d,** Change of total cloud fraction in March–April–May (MAM) (**a**), June–July–August (JJA) (**b**), September–October–November (SON) (**c**), and December–January–February (DJF) (**d**) from each individual model. Only significant values at the 90% confidence level based on a bootstrap method are shown.

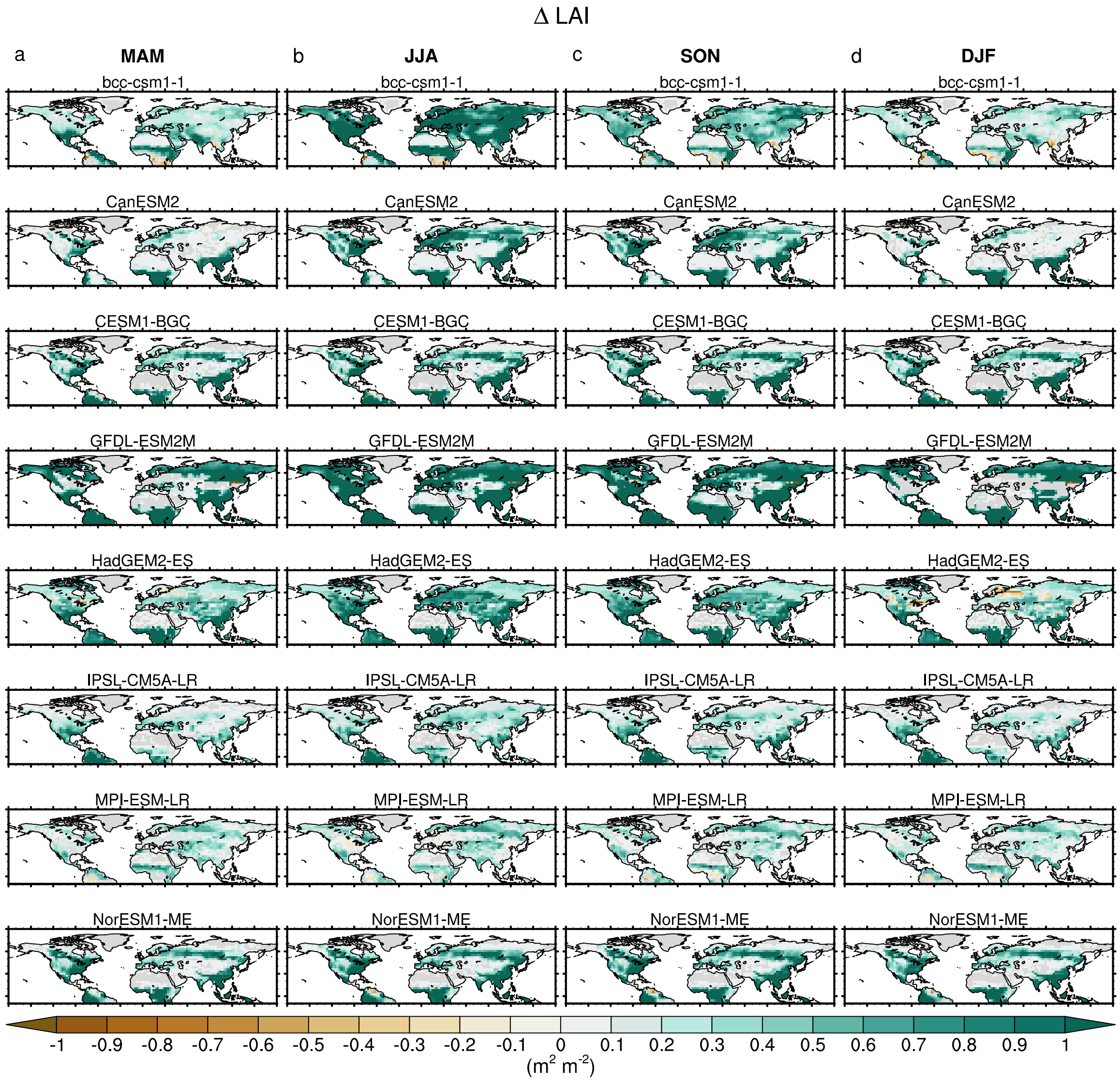


**Supplementary Figure 13 | Seasonal change in the leaf area index (LAI) resulting from $CO_2$ physiological forcing from CMIP5 ESMs. a–d,** Change of leaf area index in March–April–May (MAM) (**a**), June–July–August (JJA) (**b**), September–October–November (SON) (**c**), and December–January–February (DJF) (**d**) from each individual model. GFDL-ESM2M does not provide the LAI in 1pctCO2 simulation. Thus, the change of LAI in GFDL-ESM2M is calculated from the difference between esmFixClim1 and piControl as only $CO_2$ physiological forcing excluding a nonlinear interaction. Only significant values at the 90% confidence level based on a bootstrap method are shown.

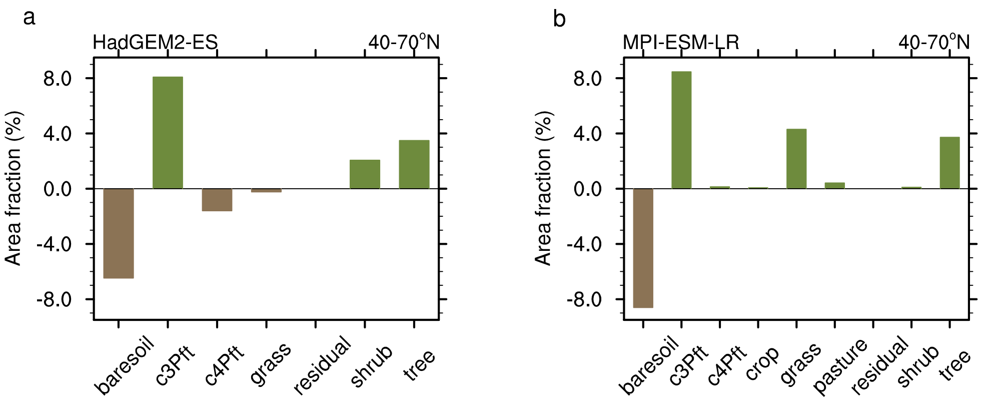


**Supplementary Figure 14 | Impacts of $CO_2$ physiological forcing on surface coverage fraction in CMIP5 ESMs coupled with DGVM. a–b,** Area-weighted average of change in annual mean fractional surface coverage resulting from $CO_2$ physiological forincg in HadGEM2-ES (**a**) and MPI-ESM-LR (**b**) in continental regions (40°–70°N). Note that the variable varies in each model, depending on their Plant Functional Type (PFT) definitions. GFDL-ESM2M does not provide the surface fractional coverage in 1pctCO2 simulation.

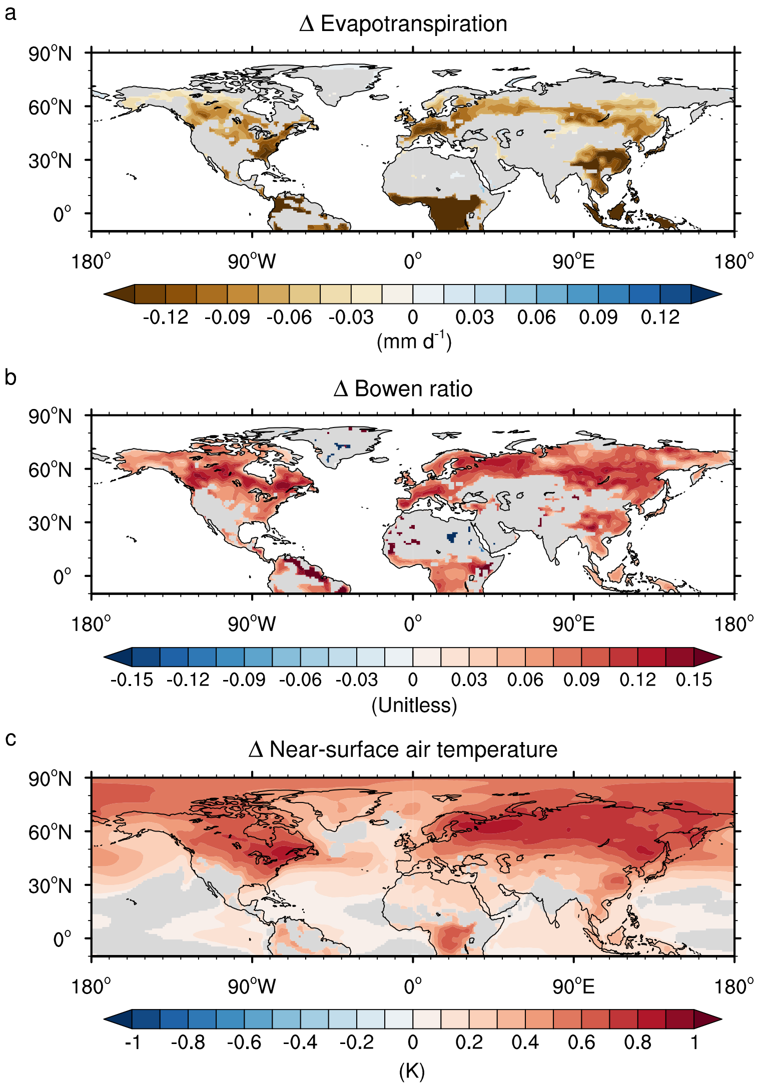


**Supplementary Figure 15 | Change in the annual mean evapotranspiration, Bowen ratio and near-surface air temperature resulting from $CO_2$ physiological forcing excluding HadGEM2-ES. a–c,** Multi-model mean change in the annual mean evapotranspiration **(a)**, Bowen ratio (sensible heat flux/latent heat flux) **(b)**, and near-surface air temperature **(c)** resulting from $CO_2$ physiological forcing excluding HadGEM2-ES. Only significant values at the 95% confidence level based on a bootstrap method are shown.

**Supplementary Table 1 | CMIP5 ESMs used in this study**

| Model Name | Modeling Center (or Group) | Reference |
|---|---|---|
| bcc-csm1-1 | Beijing Climate Center, China Meteorological Administration | (11) |
| CanESM2 | Canadian Centre for Climate Modelling and Analysis | (12) |
| CESM1-BGC | Community Earth System Model Contributors | (13) |
| GFDL-ESM2M | NOAA Geophysical Fluid Dynamics Laboratory | (14) |
| HadGEM2-ES | Met Office Hadley Centre | (15) |
| IPSL-CM5A-LR | Institute Pierre Simon Laplace | (16) |
| MPI-ESM-LR | Max Planck Institute for Meteorology | (17) |
| NorESM1-ME | Norwegian Climate Centre | (18) |

**Supplementary Table 2 | Land components of CMIP5 ESMs used in this study**

| Model Name | Dynamic Global Vegetation Model | Stomatal Conductance Scheme |
|---|---|---|
| bcc-csm1-1 | No | Ball-Berry |
| CanESM2 | No | Leuning |
| CESM1-BGC | No | Ball-Berry |
| GFDL-ESM2M | Yes (LM3V) | Leuning |
| HadGEM2-ES | Yes (TRIFFID) | Simplified Leuning |
| IPSL-CM5A-LR | No | Ball-Berry |
| MPI-ESM-LR | Yes (JSBACH) | Knorr |
| NorESM1-ME | No | Ball-Berry |

The Ball–Berry stomatal conductance scheme is based on relative humidity at the leaf surface[19] and the Leuning stomatal scheme is based on leaf-to-air vapour pressure deficit[20].

**Supplementary Table 3 | List of CMIP5 simulations used in this study**

| CMIP5 Simulations | Expansion | Description |
|---|---|---|
| 1pctCO2 (Full) | 1 percent per year $CO_2$ | Both radiation and carbon cycle see 1% per year increase in atmospheric $CO_2$ for 140 years from pre-industrial $CO_2$ concentration to quadrupling (from 285 to 1140 ppm) |
| esmFixClim1 (Physiology) | ESM fixed climate 1 | Radiation code sees pre-industrial $CO_2$ concentration, but carbon cycle sees 1%/yr $CO_2$ increase to quadrupling for 140 years (from 285 to 1140 ppm) |
| esmFdbk1 (Radiation) | ESM feedback 1 | Carbon cycle sees pre-industrial $CO_2$ concentration, but radiation code sees 1%/yr $CO_2$ increase to quadrupling for 140 years (from 285 to 1140 ppm) |
| piControl | Pre-industrial Control | Pre-industrial control run |

**Supplementary Table 4 | Changes of climate variables in mid-to-high latitudes (40°–70°N) resulting from $CO_2$ physiological forcing.**

| Variables | (Units) | Mid-to-high latitude continents (40°–70°N) | | | | |
|---|---|---|---|---|---|---|
| | | ANN | MAM | JJA | SON | DJF |
| Near-surface air temperature | (K) | 0.72 | 0.59 | 1.05 | 0.64 | 0.61 |
| Evapotranspiration | (mm $day^{-1}$) | –0.05 | –0.05 | –0.12 | –0.03 | 0.01 |
| Latent heat flux | (W $m^{-2}$) | –1.38 | –1.37 | –3.56 | –0.88 | 0.31 |
| Sensible heat flux | (W $m^{-2}$) | 2.12 | 2.26 | 5.09 | 1.13 | 0.00 |
| Relative humidity | (%) | –1.55 | –1.60 | –3.22 | –1.15 | –0.24 |
| Total cloud fraction | (%) | –0.98 | –0.84 | –2.10 | –1.10 | 0.10 |
| Downwelling SW | (W $m^{-2}$) | 1.97 | 1.50 | 5.26 | 1.31 | –0.20 |
| Upwelling SW | (W $m^{-2}$) | 0.19 | –0.87 | 0.68 | 0.03 | –0.61 |
| Downwelling LW | (W $m^{-2}$) | 2.17 | 1.40 | 3.02 | 1.93 | 2.29 |
| Upwelling LW | (W $m^{-2}$) | 3.58 | 2.85 | 5.73 | 3.13 | 2.58 |
| Net SW down | (W $m^{-2}$) | 2.17 | 2.38 | 4.58 | 1.28 | 0.42 |
| Net LW down | (W $m^{-2}$) | –1.41 | 1.45 | –2.71 | –1.19 | –0.29 |
| Snow concentration | (%) | –0.59 | –0.55 | –0.13 | –0.73 | –0.96 |

All values are area-weighted averages of eight ESMs over continents (40°–70°N) except for snow concentration, which are the averages of five ESMs (bcc-csm1-1, CanESM2, CESM-BGC, MPI-ESM-LR and NorESM1-ME). The acronyms stand for SW: shortwave flux and LW: longwave flux. Note that positive values of latent heat flux and sensible heat flux indicate upward transport. In contrast, positive values of net SW and LW down indicate downward.

**Supplementary Table 5 | Change in temperature caused by only-$CO_2$ physiological forcing (Phy-only) and by a nonlinear interaction between physiological forcing and radiative forcing (e)**.

| | ANN | MAM | JJA | SON | DJF |
|---|---|---|---|---|---|
| | Mid-to-high latitude continents (40°–70°N) | | | | |
| Phy-only | 0.57 | 0.49 | 0.87 | 0.55 | 0.39 |
| e | 0.15 | 0.10 | 0.18 | 0.09 | 0.22 |
| | Arctic region (70°–90°N) | | | | |
| Phy-only | 0.57 | 0.43 | 0.31 | 0.92 | 0.62 |
| e | 0.18 | 0.21 | 0.21 | -0.08 | 0.37 |

**Supplementary Table 6 | Changes of climate variables in Arctic region (70°–90°N) resulting from $CO_2$ physiological forcing.**

| Variables | (Units) | Arctic region (70°–90°N) | | | | |
|---|---|---|---|---|---|---|
| | | ANN | MAM | JJA | SON | DJF |
| Near-surface air temperature | (K) | 0.75 | 0.64 | 0.52 | 0.84 | 0.99 |
| Evapotranspiration | (mm $day^{-1}$) | 0.02 | 0.01 | -0.01 | 0.02 | 0.04 |
| Latent heat flux | (W $m^{-2}$) | 0.46 | 0.38 | -0.32 | 0.66 | 1.13 |
| Sensible heat flux | (W $m^{-2}$) | 0.38 | 0.08 | 0.53 | 0.10 | 0.81 |
| Total cloud fraction | (%) | 0.14 | 0.45 | –0.99 | 0.41 | 0.69 |
| Downwelling SW | (W $m^{-2}$) | –0.22 | –1.24 | 0.54 | –0.14 | –0.02 |
| Upwelling SW | (W $m^{-2}$) | –1.32 | –2.42 | –2.57 | –0.25 | –0.04 |
| Downwelling LW | (W $m^{-2}$) | 2.94 | 2.64 | 1.86 | 3.81 | 3.43 |
| Upwelling LW | (W $m^{-2}$) | 3.19 | 2.58 | 2.43 | 3.70 | 4.02 |
| Net SW down | (W $m^{-2}$) | 1.10 | 1.18 | 3.11 | 0.11 | 0.01 |
| Net LW down | (W $m^{-2}$) | –0.25 | –0.06 | –0.57 | –0.12 | 0.59 |
| Sea ice concentration | (%) | –2.28 | –1.30 | –2.34 | –2.68 | –2.81 |
| Sea ice thickness | (m) | -0.06 | -0.07 | -0.06 | -0.04 | -0.06 |

All values are area-weighted averages of eight ESMs over the Arctic region (70°–90°N). The acronyms stand for SW: shortwave flux and LW: longwave flux. Note that positive values of latent heat flux and sensible heat flux indicate upward transport. In contrast, positive values of net SW and LW down indicate downward.

## Supplementary References


1. Quillet, A., Peng, C., & Garneau, M. Toward dynamic global vegetation models for simulating vegetation–climate interactions and feedbacks: recent developments, limitations, and future challenges. *Environ. Rev.* **18**, 333–353 (2010).
2. Anav, A. et al. Evaluation of land surface models in reproducing satellite derived leaf area index over the high-latitude Northern Hemisphere. Part II: Earth system models. *Remote Sens.* **5**, 3637–3661 (2013).
3. Murray-Tortarolo, G. et al. Evaluation of DGVMs in reproducing satellite derived LAI over the Northern Hemisphere. Part I: Uncoupled DGVMs. *Remote Sens.* **5**, 4819–4838 (2013).
4. Brovkin, V. et al. Evaluation of vegetation cover and land-surface albedo in MPI-ESM CMIP5 simulations. *J. Adv. Model. Earth Sy.* **5**, 48–57 (2013).
5. Mahowald, N. et al. Projections of leaf area index in earth system models. *Earth Syst. Dyn.* **7**, 211–229 (2016).
6. Medlyn, B. E. et al. Effects of elevated [$CO_2$] on photosynthesis in European forest species: a meta-analysis of model parameters. *Plant. Cell. Environ*. **22**, 1475–1495 (1999).
7. Keel, S. G., Pepin, S., Leuzinger, S., & Körner, C. Stomatal conductance in mature deciduous forest trees exposed to elevated $CO_2$. *Trees* **21**, 151 (2007).
8. Damour, G., Simonneau, T., Cochard, H., & Urban, L. An overview of models of stomatal conductance at the leaf level. *Plant. Cell. Environ*. **33**, 1419–1438 (2010).
9. Paschalis, A., Katul, G. G., Fatichi, S., Palmroth, S., & Way, D. On the variability of the ecosystem response to elevated atmospheric $CO_2$ across spatial and temporal

scales at the Duke Forest FACE experiment. *Agric. For. Meteorol.* **232**, 367–383 (2017).

10. Fatichi, S. et al. Partitioning direct and indirect effects reveals the response of water-limited ecosystems to elevated $CO_2$. *Proc. Natl Acad. Sci. USA* **113**, 12757–12762 (2016).
11. Wu, T. et al. Global carbon budgets simulated by the Beijing Climate Center Climate System Model for the last century. *J. Geophys. Res. Atmos.* **118**, 4326–4347 (2013).
12. Arora, V. K. et al. Carbon emission limits required to satisfy future representative concentration pathways of greenhouse gases. *Geophys. Res. Lett.* **38**, L05805 (2011).
13. Lindsay, K. et al. Preindustrial-control and Twentieth-century Carbon cycle experiments with the earth system model CESM1(BGC). *J. Clim.* **27**, 8981–9005 (2014).
14. Dunne, J. P. et al. GFDL's ESM2 global coupled climate–carbon earth system models. Part I: Physical formulation and baseline simulation characteristics. *J. Clim.* **25**, 6646–6665 (2012).
15. Jones, C. D. et al. The HadGEM2-ES implementation of CMIP5 centennial simulations. *Geosci. Model Dev.* **4**, 543–570 (2011).
16. Dufresne, J. L. et al. Climate change projections using the IPSL-CM5 Earth System Model: from CMIP3 to CMIP5. *Clim. Dyn.* **40**, 2123–2165 (2013).

17. Giorgetta, M. A. et al. Climate and carbon cycle changes from 1850 to 2100 in MPI-ESM simulations for the Coupled Model Intercomparison Project phase 5. *J. Adv. Model. Earth Syst.* **5**, 572–597 (2013).

18. Tjiputra J. F. et al. Evaluation of the carbon cycle components in the Norwegian Earth System Model (NorESM). *Geosci. Model Dev.* **6**, 301–325 (2013).

19. Ball, J. T., Woodrow, I. E. & Berry, J. A. In *Progress in Photosynthesis Research, Vol. 4. Proceedings of the VIIth International Congress on Photosynthesis. Providence, Rhode Island, USA, August 10–15, 1986* (ed. Biggins, J.) 221–224 (Springer, Netherlands, 1987).

20. Leuning, R. A critical appraisal of a combined stomatal-photosynthesis model for C3 plants. *Plant, Cell & Environ.* **18,** 339–355 (1995).
